# Global Repertoire of Human Antibodies Against *Plasmodium falciparum* RIFINs, SURFINs, and STEVORs in a Malaria Exposed Population

**DOI:** 10.3389/fimmu.2020.00893

**Published:** 2020-05-12

**Authors:** Bernard N. Kanoi, Hikaru Nagaoka, Michael T. White, Masayuki Morita, Nirianne M. Q. Palacpac, Edward H. Ntege, Betty Balikagala, Adoke Yeka, Thomas G. Egwang, Toshihiro Horii, Takafumi Tsuboi, Eizo Takashima

**Affiliations:** ^1^Division of Malaria Research, Proteo-Science Center, Ehime University, Matsuyama, Japan; ^2^Department of Parasites and Insect Vectors, Pasteur Institute, Paris, France; ^3^Department of Malaria Vaccine Development, Research Institute for Microbial Diseases, Osaka University, Suita, Japan; ^4^Department of Plastic and Reconstructive Surgery, Graduate School of Medicine and Hospital, University of the Ryukyus, Okinawa, Japan; ^5^Department of Tropical Medicine and Parasitology, School of Medicine, Juntendo University, Tokyo, Japan; ^6^Makerere University School of Public Health, Kampala, Uganda; ^7^Med Biotech Laboratories, Kampala, Uganda

**Keywords:** *Plasmodium falciparum*, RIFIN, STEVOR, SURFIN, naturally acquired immunity

## Abstract

Clinical immunity to malaria develops after repeated exposure to *Plasmodium falciparum* parasites. Broadly reactive antibodies against parasite antigens expressed on the surface of infected erythrocytes (variable surface antigens; VSAs) are candidates for anti-malaria therapeutics and vaccines. Among the VSAs, several RIFIN, STEVOR, and SURFIN family members have been demonstrated to be targets of naturally acquired immunity against malaria. For example, RIFIN family members are important ligands for opsonization of *P. falciparum* infected erythrocytes with specific immunoglobulins (IgG) acquiring broad protective reactivity. However, the global repertoire of human anti-VSAs IgG, its variation in children, and the key protective targets remain poorly understood. Here, we report wheat germ cell-free system-based production and serological profiling of a comprehensive library of A-RIFINs, B-RIFINs, STEVORs, and SURFINs derived from the *P. falciparum* 3D7 parasite strain. We observed that >98% of assayed proteins (*n* = 265) were immunogenic in malaria-exposed individuals in Uganda. The overall breadth of immune responses was significantly correlated with age but not with clinical malaria outcome among the study volunteers. However, children with high levels of antibodies to four RIFINs (PF3D7_0201000, PF3D7_1254500, PF3D7_1040600, PF3D7_1041100), STEVOR (PF3D7_0732000), and SURFIN 1.2 (PF3D7_0113600) had prospectively reduced the risk of developing febrile malaria, suggesting that the 5 antigens are important targets of protective immunity. Further studies on the significance of repeated exposure to malaria infection and maintenance of such high-level antibodies would contribute to a better understanding of susceptibility and naturally acquired immunity to malaria.

## Introduction

Acquired clinical immunity, such as protective immunity against febrile malaria, develops progressively after repeated exposure to *Plasmodium falciparum* (1). During cyclic intra-erythrocytic development, the parasite exports numerous proteins to the surface of infected red blood cells (iRBCs). These exported proteins are collectively referred to as variable surface antigens (VSAs) and include *P. falciparum* erythrocyte membrane protein 1 (PfEMP1), surface-associated interspersed gene family (SURFIN), repetitive interspersed family (RIFIN) proteins, and subtelomeric variable open reading frame (STEVOR) proteins (2–6).

In the reference *P. falciparum* 3D7 genome, RIFIN is the largest multigene family and is encoded by ~180 *rif* genes (7). Multiple RIFINs are simultaneously expressed 11–12 h post RBC invasion (8) and are subsequently exported to the surface of iRBCs. Some RIFIN variants are expressed in other parasite stages such as merozoites and sporozoites (9, 10). STEVORs, encoded by 30 *stevor* genes in the 3D7 reference strain, are structurally related to RIFINs; specifically, they share minimal sequence similarity, a predicted signal peptide at the N-terminus, a PEXEL motif, an N-terminal conserved region, a central hydrophobic region, a variable stretch, a transmembrane domain, and a short, charged C-terminal segment (11). The membrane topologies of the RIFIN and STEVOR protein families are central considerations of their function. Some early models of RIFIN and STEVOR structure favored a two transmembrane topology (12), but the transmembrane prediction tool TMHMM (13, 14) and recent experimental data indicate a central hydrophobic stretch that may interact with the external face of erythrocyte membrane, a single C-terminal transmembrane domain (15, 16), and an N-terminal extracellular host ligand-binding domain (17, 18). The C-terminal variable region is found between the central hydrophobic region and the transmembrane domain for all RIFINs and STEVORs.

RIFINs in the 3D7 strain can be subdivided into two major groups: A- and B-RIFINs. The difference is largely due to a 25 amino acid indel present only in the conserved N-terminal region of A-RIFINs (19). Immunofluorescence studies to distinguish the subtypes suggest that during the intraerythrocytic stage A-RIFINs are expressed on the infected erythrocyte surface and merozoite apical region while B-RIFINs are retained in the parasite cytosol (9). However, variant-specific B-RIFIN antibodies, at least for PF3D7_1300600, suggest they could also be expressed on the surface of merozoites (20). The role of B-RIFINs as targets of protective immunity against clinical malaria remains unclear.

We recently demonstrated that *P. falciparum* parasites express numerous RIFINs to evade host immunity through targeting inhibitory receptors (2). Specifically, iRBCs from patients with severe malaria were observed to often display human leucocyte immunoglobulin-like receptor B1 (LILRB1)-binding RIFINs, that contributed to the pathogenesis of the disease (2). Other studies reported RIFINs as targets of naturally acquired immunity to severe malaria (21, 22). It has also been observed that increased antibody titers against certain RIFINs are associated with prompt parasite clearance and suppression of malaria symptoms in Gabonese children (23). Furthermore, specific IgGs that acquire broad protective reactivity against RIFIN family members may prevent RIFIN binding to host leucocytes (2) and support opsonization of *P. falciparum* infected erythrocytes (24), hence limiting disease progression.

The role of STEVORs in malaria immunity remains underexplored. However, the expression of these proteins in multiple parasite stages including sporozoites (4), trophozoites (2, 3), schizonts/merozoites (3, 6, 15), and gametocytes (25) suggests that the proteins mediate multiple important functions in parasite development. Given the structural similarity of STEVORs and RIFINs, together with their host cell surface expression (16), the two families may have related functions in immune evasion and/or protective immunity. Indeed, lack of immunity to distinct STEVORs has been associated with vulnerability to severe malaria in Malian children (22). Likewise, *in vitro* experiments demonstrated that anti-STEVOR antibodies not only interrupt merozoite attachment to the erythrocyte and effectively inhibit RBC invasion (15, 26), but also contribute to the rigidity of infected erythrocytes and rosette formation with non-infected cells (15, 17, 26).

Unlike the large gene families of STEVORs and RIFINs, in Pf3D7 SURFINs are represented by a small family of 10 *surf* genes which encode large proteins (200–300 kDa) (5). The SURFINs have a cysteine-rich predicted extracellular globular domain, single transmembrane domain, a large cytoplasmic domain containing tryptophan-rich repeats, and lack apparent signal peptide sequences (5, 27). SURFIN members exhibit stage-specific expression during the parasite life cycle. For example, while the expression of *surf1.1, surf4.1*, and *surf14.1* appear to be restricted to late trophozoites and/or during schizont development, *surf1.3, surf4.2*, and *surf8.3* are expressed throughout the erythrocytic cycle (28). At least for SURFIN 4.2, the protein is under host immune pressure due to localization on the surface of merozoites and infected erythrocytes (28, 29).

Numerous studies have shown that individuals residing in malaria endemic areas slowly and gradually acquire age-dependent immunity to clinical malaria. This is evidenced by age-associated decreases in cases of severe and uncomplicated malaria episodes in the population (1). It has long been noted that antibodies are key effector molecules in this immunity, since passive transfer of IgG from malaria immune adults to children with malaria alleviated clinical symptoms and induced a steep reduction in parasitemia (30). During infection, numerous *P. falciparum* proteins interact with host immunity inducing an array of potentially protective antibodies hence making them important targets of naturally acquired immunity (31, 32). Until recently, concerted efforts to study VSAs have focused mainly on the role of anti-PfEMP1 immunity in blocking vascular adhesion and sequestration of iRBCs [reviewed in Bull and Abdi (33)]. However, the global repertoire of human antibodies against the VSA SURFINs, RIFINs, and STEVORs, and their association with protective immunity against clinical malaria remains unevaluated. VSAs could be useful targets for therapeutics and vaccines against malaria, but advances in validating their usefulness have been hampered by the difficulty in expressing parasite AT-rich genes. In an attempt to overcome this challenge, this study employed the robust and high-throughput eukaryotic wheat germ cell-free protein synthesis system (WGCFS) coupled with a homogeneous high-throughput AlphaScreen system for antibody profiling (34–36); to comprehensively assess antibody responses to RIFINs, STEVORs, and SURFINs in a cohort of malaria exposed children and young adults in Uganda. The study allowed us to assess immune responses to a large panel of malaria proteins to identify potential targets of protective antibodies in clinical malaria.

## Materials and Methods

### Study Setting and Ethical Statement

Serum samples used in this study were obtained from residents of Lira Municipality in Northern Uganda, a region characterized by high malaria transmission (37–41). *Anopheles fenestus* is the principal malaria vector (41). Sixty-six healthy study participants aged 6–20 years were enrolled in the control arm (non-vaccinated control group) of a malaria vaccine follow-up study (37).

Permission to use the samples and study protocols were approved by the Institutional Research and Ethics Committee of Lacor Hospital (LHIREC 023/09/13), Uganda National Council for Science and Technology (HS1403) in Uganda; and Ethics Committees of the Research Institute for Microbial Diseases, Osaka University and Ehime University. Written informed consent was obtained from all study participants and/or their parents or guardians before the study. Verbal and written assent was obtained from children aged 6–7 years and 8–17 years, respectively. The assent took precedent over consent from the parents or guardians. The study was conducted in compliance with the International Conference on Harmonization, Good Clinical Practices and the Declaration of Helsinki.

### Production of a *P. falciparum* Parasite Protein Library

**Figure 1:**
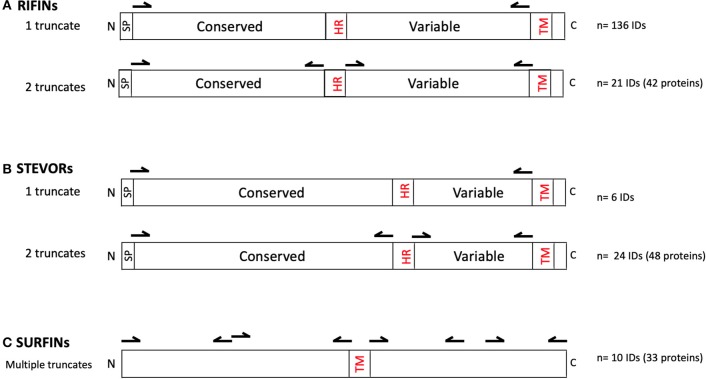
Primary structures of **(A)** RIFIN, **(B)** STEVOR, and **(C)** SURFIN families. Illustrated are conserved and variable domains, signal peptide (SP), central hydrophobic region (HR), transmembrane domains (TM), N-terminus (N), and C-terminus **(C)**. Arrows indicate the approximate DNA priming regions for creating the truncated proteins excluding SP, HR, and TM. As indicated, 136 *rif* and 6 *stevor* genes were designed to be expressed as one truncate; and 21 *rif* and 24 *stevor* genes as two truncates each. The resultant number of recombinant proteins were: RIFINs *n* = 178 (from 157 unique IDs); STEVORs, *n* = 54 (from 29 unique IDs); and SURFINs, *n* = 33 (from 10 unique IDs). The region and number of truncates derived from each gene in the VSA family are detailed in Table S1.

The RIFINs, STEVORs, and SURFINs were expressed as described (36) using sequences retrieved from PlasmoDB derived from the Pf3D7 reference strain. All proteins were hand-curated using SignalP 5 (27) and TMHMM-2.0 (13, 14) to locate potential signal peptides and transmembrane domains, respectively. Central hydrophobic regions were also identified. To ease recombinant protein expression using the WGCFS, the protein truncates were designed such that they possessed an ecto-domain but lacked the signal peptide, central hydrophobic domain, as well as the C-terminal transmembrane domain and charged segment (as reflected in Table S1 and illustrated in Figure 1). Specifically, 136 *rif* and 6 *stevor* genes were designed to be expressed as one truncate (Table S1a). An additional 21 *rif* and 24 *stevor* were designed for two truncates corresponding to the N-terminal (conserved) or variable C-terminal regions after removing the central hydrophobic region and C-terminal transmembrane domain (Table S1b). Due to their large sizes, SURFINs were designed to be expressed as 3–4 truncates with an average of ~ 600 aa each, but excluding potentially hydrophobic regions where present (e.g., in PF3D7_1301800) and transmembrane domains (Table S1c).

DNA sequences were amplified using PrimeSTAR Max DNA polymerase (Takara Bio, Kusatsu, Japan) and region-specific primers (Table S1) and cloned into the pEU vector (CellFree Sciences, Matsuyama, Japan) using In-Fusion HD Cloning Kit (Takara Bio). The pEU vector is specifically designed for the WGCFS protein expression platform. DNA transcription and WGCFS protein synthesis were semi-automated using a GenDecoder1000 robotic protein synthesizer (CellFree Sciences). Proteins were expressed as N-terminus mono-biotinylated recombinant proteins by inclusion of 50 ng biotin-protein ligase (BirA) in the translation lower layer, and adding 500 nM D-biotin (Nacalai Tesque, Kyoto, Japan) to both the translation and substrate upper layers. The expressed recombinant proteins were assessed using Western blot by probing with HRP conjugated streptavidin (42). The constructed recombinant protein library consisted of RIFINs *n* = 178 (from 157 unique IDs); STEVORs, *n* = 54 (from 29 unique IDs); and SURFINs, *n* = 33 (from 10 unique IDs). RIFINs can be sub-grouped as A-RIFINs (*n* = 120), B1-RIFINs (*n* = 15), B2-RIFINs (*n* = 17), and other B-types (*n* = 26) according to published work (Table S1) (11).

### Quantitation of Parasite Specific Antibodies by AlphaScreen

Human serum antibody levels against the RIFIN, STEVOR, and SURFIN protein library were quantified by AlphaScreen as described (35, 42). Briefly, biotinylated recombinant *P. falciparum* proteins were dispensed into a 384-well OptiPlate using a JANUS Automated Workstation dispenser (PerkinElmer, Waltham, MA) and mixed with 10 μl of 4000-fold diluted sera in reaction buffer (100 mM Tris-HCl [pH 8.0], 0.01 % [v/v] Tween-20, and 0.1 % [w/v] bovine serum albumin). After 30 min incubation at 26°C, 10 μl of detection mixture containing streptavidin-coated donor-beads (PerkinElmer) and protein G (Thermo Scientific, Waltham, MA) conjugated acceptor-beads (PerkinElmer) was added, to make a final concentration of 12 μg/ml for both beads. The plate was then incubated in the dark for 1 hr at 26°C to allow optimal binding of the donor and acceptor-beads to the biotinylated protein and human antibody, respectively. Luminescence emitted by acceptor-beads upon excitation of the donor-beads was detected using an EnVision plate reader (PerkinElmer) and captured as raw AlphaScreen Counts (ASC_raw_). To account for day-to-day and plate-to-plate assay variability, serially diluted biotinylated rabbit IgG (PerkinElmer) was included in each plate, and subsequently used to generate a 5-parameter logistic standardization curve. The assays were randomized to minimize experimental bias.

### Statistical Analysis

Data analysis was performed using either R software (Version 3.5.2 “Eggshell Igloo”; R Foundation for Statistical Computing) or Prism 6 (GraphPad Software Inc., La Jolla, CA).

The cut off point for protein seropositivity to human serum was set at half the lowest non-negative ASC value from that of the assayed samples (43), and a protein was deemed immunoreactive if more than 10% of the volunteers had ASC levels above the seropositivity cut-off. Due to differences in the yield of individual expressed proteins, immunoreactivity was compared among the human serum samples but not across different proteins.

Overall survival was defined as the time from first sampling, when all individuals enrolled were blood-smear negative (37), to the first clinical malaria episode. For this study, a febrile malaria episode was defined as having fever ≥37.5°C and asexual parasitemia of ≥2,500/μl of blood but with no sign of complicated disease. Although some children had multiple parasitic or febrile malaria episodes, only the first symptomatic episode was considered. Analysis based on multiple episodes was precluded because of low incidence. For analysis on the effects of an individual antigen, individuals were categorized as “High Responders” (with an ASC value above the population median for that antigen) or “Low Responders.” Kaplan–Meier curves were created to compare survival between groups, and the log-rank statistic was used to test the hypothesis of a difference in survival between groups (high responders vs. low-responders). Cox proportional hazards models were used to calculate hazard ratios (HR) and 95% confidence intervals (CIs) to assess whether the presence of antigen specific antibodies was associated with overall survival. A similar approach has been used in other studies of this type (35, 44–46). Potential protective efficacy (PPE) was computed as 1 − hazard ratio (PPE% = (1 − HR) ×100%). Associations between age and clinical outcome were examined by the Mann–Whitney *U*-test or Kruskal–Wallis with Bonferroni–Dunn as a *post hoc* test.

Multivariate survival analysis while adjusting for confounders focused on bed-net use and age as a categorical variable (6–10 years, 11–15 years, and 16–20 years). Analysis while adjusting for different antibody responses as covariates was limited by their highly correlated nature. All tests were two-sided. A *P*-value <0.05 was considered significant, and the values presented here were not adjusted for multiple comparisons, rather the interpretation of associations is based on the level of significance and direction of the association between antibody responses and clinical malaria as the primary outcome. Sensitivity survival analysis was conducted using the overall survival outcome of protection (time to first malaria episode) but only with samples obtained from individuals (*n* = 52) with at least one microscopically confirmed parasitic infection during the follow up period (47, 48). Modified Poisson Regression Model for prospective study with binary outcome associating High Responders vs. Low Responders was also conducted.

## Results

WGCFS coupled with AlphaScreen is optimal for determining levels of antibodies that interact with linear and conformational epitopes. Here, we assessed antibody responses against a library of all RIFINs, SURFINs, and STEVORs derived from the Pf3D7 strain. We probed the library with 66 sera from Ugandan individuals aged 6–20 years to identify seroreactive domains/regions that are protective against febrile malaria.

### Seroreactivity and Seroprevalence of RIFINs, SURFINs, and STEVORs

**Figure 2:**
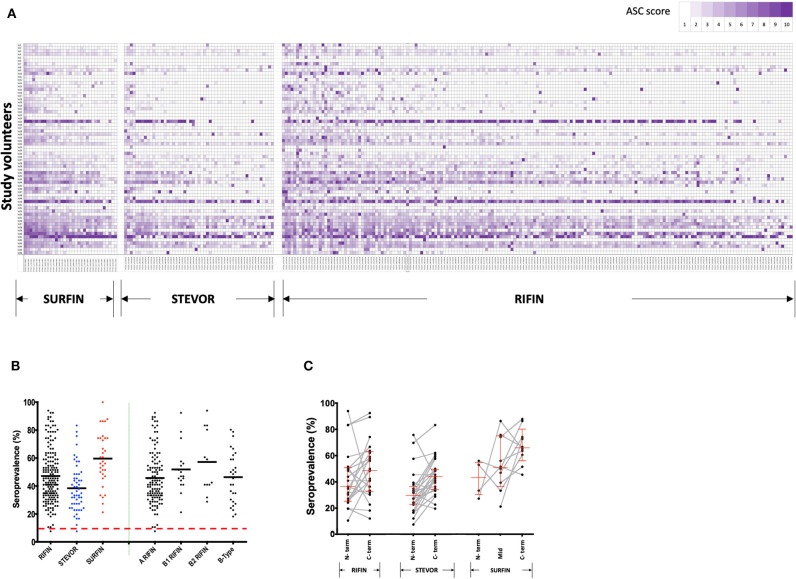
Immunoreactivity and seroprevalence of antibodies to RIFINs, STEVORs, and SURFINs. **(A)** Global profiling of anti-VSA antibodies. Antibodies against variable surface antigens in Ugandans (*n* = 66) aged 6–20 years. The proteins recognized in each VSA family are indicated at the bottom. The serum samples were sorted by age from top (6 years) to bottom (20 years). The antigens are ordered from those with highest mean ASC score (left) to lowest mean ASC score (right). To generate the heatmap, ASC scores (range 1–10) for each antigen were generated based on ASC_raw_, where 10 represent the highest ASC_raw_ and 1 represent the lowest ASC_raw_. **(B)** Antibody immunoreactivity to RIFINs, STEVORs, and SURFINs are presented by black, blue, and red dots, respectively. The right side of the plot shows seroprevalence of difference RIFIN family sub-groups. The bold horizontal line in each group denotes the overall median per group. There was no difference in the seroprevalence across protein families (*P* > 0.05). The dashed red horizontal line indicates the protein immunoreactivity cut-off point, set at 10% seroprevalence. **(C)** Proportion of individuals recognizing proteins based on truncations specific to conserved or variable regions. The lines and markers in red show median seroprevalence with 95% CI. The N- and C-term denotes seroprevalence of recombinant proteins derived from the N- and C-terminus of the same gene, respectively. Mid represents proteins derived from the central part of the protein. Fragments of a single protein are linked. There was no difference in the seroprevalence between N- and C- terminal regions in any of the protein families (*P* > 0.05).

Antibody reactivity to the VSA library (Table S1) of RIFINs (*n* = 178), STEVORs (*n* = 54), and SURFINs (*n* = 33) (as illustrated in Figure 1) was measured at the beginning of the study, prior to the rainy season. The pattern of antibody profiles varied considerably among the volunteers. An age-dependent acquisition antibody trend could be observed (Figure S1A) but visual inspection also suggested that there are different patterns of age-related acquisitions to each protein family (Figure 2A). Antibody responses to the different antigens were highly correlated (Figure S1B).

Immunoreactivity was 99, 98, and 100% for RIFINs, STEVORs, and SURFINs, respectively. The average seroprevalence for SURFINs was 60, 48% for RIFINs, and 40% for STEVORs, suggesting that these proteins are naturally immunogenic (Figure 2B). The high immunoreactivity could also be an outcome of cross-reactivity of antibodies targeting the conserved region within the protein families. RIFINs can be subdivided into A- and three classes of B-RIFIN proteins largely due to a 25 amino acid indel present only in the conserved region of A-RIFINs (19). Based on Figure 2B, however, there was no clear distinction in the seroreactivity of RIFINs according to the subclasses A, B1, B2, or B-type. The proportion of individuals recognizing a protein based on whether it was derived from mainly the N-terminal (conserved) or variable C- terminal region of the protein showed a trend, but no significant difference within or between the protein families (Figure 2C).

**Figure 3:**
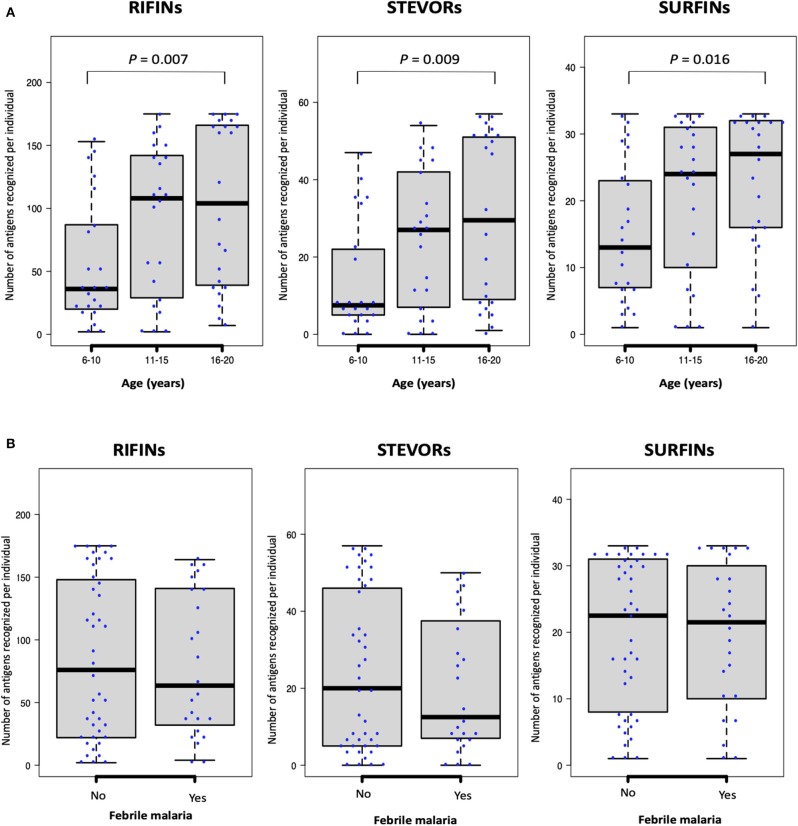
The number of antigenic targets recognized by different age-groups at study enrollment. The number of seropositive RIFINs, STEVORs, and SURFINs for each individual is shown. Box-plots indicate the median and interquartile range for each group. **(A)** As categorized by age: Statistical difference was assessed using the Kruskal–Wallis test (*P* <0.001); with Dunn's test as *post hoc* test, *P* <0.01 between age 6–10 and 16–20 years for all protein families. **(B)** As categorized by febrile malaria outcome during the follow-up period. There was no statistical difference between individuals who developed febrile malaria and those who did not develop malaria.

Generally, as is shown in Figure 2A, antibody responses to several antigens were acquired early in life with an IgG repertoire comparable between protein families. We observed a weak but significant correlation between age (years) and the number of antigens recognized per individual for each of the protein families: RIFINs, spearman's *r* = 0.3, *P* = 0.004; STEVORs, *r* = 0.3, *P* = 0.005; and SURFINs *r* = 0.3 and *P* = 0.014. When the number of recognized antigens was plotted by age group, sera from young adults (16–20 years-old) recognized significantly more antigens than sera from children (6–10 years-old, Mann–Whitney *U*-test, RIFINs, *P* = 0.007; STEVORs, *P* = 0.009; and SURFINs, *P* = 0.016; Figure 3A). The degree of antigen recognition was not significantly different between 6–10 and 11–15 years-old; or between 11–15 and 16–20 years-old. When this degree of recognition was assessed against febrile malaria events recorded during the follow-up period, the breadth of the immune response did not significantly (*P* = 0.14) associate with clinical malaria outcome to any of the VSA family (Figure 3B).

### Relationship Between Antibodies and Incidence of Febrile Malaria

**Figure 4:**
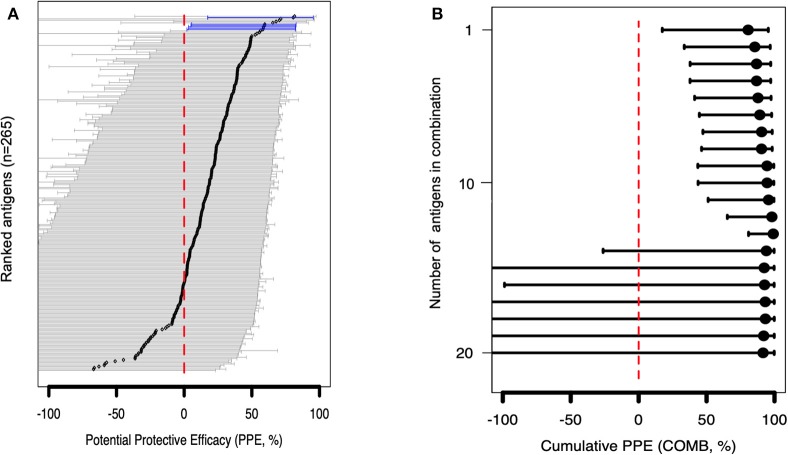
Associations between antibody levels and risk of *P. falciparum* febrile malaria. Complete list of the data for potential protective efficacy (PPE), and 95% confidence interval, is provided in Table S2. **(A)** Unadjusted association between antibody levels and risk of *P. falciparum* febrile malaria for each of the antigens tested in the cohort. Antigens are ranked—top to bottom—by the strength of their PPE. PPE (percentage) for each antigen was derived from the hazard ratio (HR) calculated by the unadjusted Cox-regression hazard model analysis [comparing children with high (High Responders) vs. low (Low Responders) antibody responses]. Black dots indicate the PPE (%), and the error bars indicate the 95% confidence interval with statistically significant PPEs represented by blue error bars. The red vertical line represents a PPE of 0% (i.e., HR = 1). **(B)** Unadjusted association between antibody levels to an increasing number of antigens—up to 20 antigens—and risk of *P. falciparum* febrile malaria. Cumulative PPE (COMB, %) represents potential protective efficacy derived from inclusion of multiple antigens in the Cox analysis model.

To gain further insight into VSA family reactivity and malaria incidence, the above results were leveraged for insights based on available data on the timing of malaria incidences. In the time-to-event univariate analysis, antigen-specific antibodies to 4 RIFINs, a SURFIN, and a STEVOR associated with protection. Specifically, immune responders to B RIFINs (PlasmoDB ID: PF3D7_0201000, PF3D7_1254500, PF3D7_1040600, PF3D7_1041100), STEVOR (PF3D7_0732000), and SURFIN 1.2 (PF3D7_0113600) had a significantly reduced risk of having febrile malaria (unadjusted Hazard ratio (HR) <1, *P* <0.05; Figure 4A and Table 1). To address inconsistency associated with heterogeneity in malaria incidence, we performed additional alternative survival analyses using the standard outcome of protection (time to first malaria episode) using only samples obtained from individuals (*n* = 52) with at least 1 microscopically confirmed parasite infection during the follow up period (47, 48). All antigens selected by primary analysis were again selected suggesting that these antigens are potentially important targets of malaria protective immunity. The trend was also similar when the Modified Poisson Regression Model was applied. Results of alternative analyses are summarized in Table S2.

**Table 1:** Top 5 antigens selected in each VSA family.

								**Unadjusted PPE^†^**				**Age and Bed-net use adjusted PPE**			
**Product ID**	**Gene ID**	**Subtype**	**Number of TM domains**	**Signal peptide**	**Protein length**	**Expressed region^**(aa)**^**	**Seroprevalence (%)**	**PPE (%)**	**25% CI**	**95% CI**	*P-*value*	**PPE (%)^**†**^**	**25% CI**	**95% CI**	***P-*value**
**RIFIN**															
RS23	PF3D7_0201000	B-type	1	Yes	312	21–113	27.27	81	17	95	**0.0265**	(178.72)	(1,404.28)	48.36	0.233
RS189	PF3D7_1254500	B-type	1	Yes	306	21–269	66.67	60	06	83	**0.0366**	(172.68)	(1,427.32)	51.32	0.254
RS158	PF3D7_1040600	B-type	1	Yes	326	28–283	75.76	59	3	82	**0.0422**	(178.19)	(1,454.72)	50.22	0.244
RS163	PF3D7_1041100	B1-type	1	Yes	334	24–291	74.24	59	3	82	**0.0422**	(186.15)	(1,487.80)	48.43	0.229
RS89	PF3D7_0700200	A-type	1	Yes	323	23–280	27.27	68	−8	90	0.0657	(240.24)	(1,789.11)	38.72	0.161
**STEVOR**															
RS99	PF3D7_0732000	STEVOR	1	Yes	296	198–256	83.33	58	2	82	**0.0447**	(209.51)	(1,581.40)	43.03	0.191
RS186	PF3D7_1254300	STEVOR	1	Yes	302	23–177	16.67	82	−37	98	0.0979	(198.98)	(1,519.75)	44.81	0.204
RS233	PF3D7_1479900	STEVOR	1	Yes	304	202–264	43.94	52	−15	80	0.0982	(211.76)	(1,593.56)	42.61	0.188
RS92	PF3D7_0700400	STEVOR	1	Yes	300	24–174	18.18	66	−47	92	0.1496	(208.04)	(1,568.66)	43.14	0.192
RS13	PF3D7_0115400	STEVOR	1	Yes	301	23–178	31.82	49	−37	81	0.1815	(220.36)	(1,662.68)	41.78	0.181
**SURFIN**															
RS264	PF3D7_0113600	SURFIN 1.2 ^#^	0	No	1893	636–1270	100.00	59	5	83	**0.0378**	(257.84)	(1,913.01)	36.39	0.148
RS242	PF3D7_1477600	SURFIN 14.1	1	No	1953	905–1442	74.24	47	−21	77	0.1294	(196.91)	(1,559.65)	46.88	0.215
RS253	PF3D7_0424400	SURFIN 4.2	1	No	2380	1195–1796	60.61	45	−25	76	0.1512	(197.13)	(1,557.40)	46.73	0.214
RS256	PF3D7_0402200	SURFIN 4.1^#^	0	No	2219	1605–2219	65.15	45	−26	76	0.1560	(205.19)	(1,625.60)	46.02	0.207
RS238	PF3D7_1301800	SURFIN 13.1^#^	1	No	2164	658–1319	50.00	−66	−2.75	26	0.2191	(259.91)	(1,870.19)	34.25	0.140

TM domains, transmembrane domains. ^aa^Amino acids. ****PPE, potential protective efficacy derived from the cox analysis hazard ratios. *In bold indicates statistically significant unadjusted PPE. No PPE remained statistically significant after adjustments for confounders (age and bed-net use). ^#^Genes annotated as pseudogenes in PlasmoDB release 41.

In the multivariable adjusted survival analysis, the correlation between antibodies and risk of febrile malaria was generally reduced but none of the antigens remained statistically significant after adjusting for age [which was reported as significantly associated with clinical malaria outcome (35)], and bed-net use (Table S2). There was a strong collinearity between the different antibody titers which limited analysis to different antibody responses as covariates (Figure S1B). Unadjusted association between antibody levels to a combination and increasing number of antigens, and risk of *P. falciparum* febrile malaria showed reduced risk for the top 13 antigens (Table S3) followed by an increased uncertainness as shown by widening confidence interval (Figure 4B), probably due to the limited number of samples assessed.

## Discussion

This study was conducted to evaluate immune responses to RIFINs, SURFINs, and STEVORs in residents of a malaria endemic region in Uganda. We assessed associations between global antibody responses to three VSA families and febrile malaria as an outcome in a follow up study. Antibody responses targeting 4 RIFINs, a SURFIN, and a STEVOR were associated (unadjusted) with reduced risk to febrile malaria suggesting that they are potential mediators in the development of uncomplicated malaria or are targets of naturally acquired immunity against clinical malaria.

Residents in malaria endemic regions acquire immunity to severe malaria early in life before the acquisition of clinical immunity. Thus, it has been proposed that antibodies against VSAs such as RIFINs, SURFINs, and STEVORs that are associated with severe malaria are acquired in early childhood. However, as shown in this study, not all VSAs present in elder groups could be detected in young children (Figure 2A) suggesting that only a limited set of specific VSAs could be involved in protection against severe malaria in early childhood. The antibody reactivity against the Pf3D7 derived RIFINs, SURFINs, and STEVORs as observed here demonstrate that individuals exposed to natural infection develop antibodies that recognize VSAs expressed by heterologous parasites and not necessarily only the parasite causing the clinical episode. Although a certain level of cross-reactivity may be argued despite the variability of the tested proteins, a similar observation has also been made against PfEMP1 and RIFINs in controlled human infection studies in malaria naïve individuals (49). The findings could also suggest that sequence variability does not affect the structure and functionality of VSAs (50, 51). One question that must be asked and that requires further investigation is whether these VSAs could potentially be targets of effective anti-disease vaccines.

The RIFIN family has recently been identified as an important ligand for opsonization of iRBCs, with specific antibodies acquiring broad reactivity through a novel mechanism of insertion of a large DNA fragment between the V and DJ segments (24). Based on this background, the identification of 4 RIFINs here highlights the potential use of RIFINs as targets for passive and active inducers of protective immunity. Specifically, antibody levels to several RIFIN proteins have been shown to correlate with parasite clearance, abrogation of symptoms of febrile malaria among Gabonese children (23), and association with severe malaria in Tanzanian patients (2). Although it is still unknown how naturally acquired antibodies to RIFIN function, recent *in vitro* studies suggest that the antibodies act by disrupting rosette formation around iRBCs, promoting phagocytosis, and blocking the binding of RIFINs to host B- and NK immune cells immunoglobulin-like receptor B1 (LILRB1) (2, 24, 29). Furthermore, RIFIN specific LAIR1-containing antibodies have been observed to potentiate agglutination and opsonization, consistent with a role of LAIR1-containing antibodies in decreasing the burden of iRBCs *in vivo* by enhancing parasite clearance. However, due to the clonal expression of RIFIN proteins (52), anti-RIFIN antibodies alone may not be sufficient to take full control of the infection. This would suggest that the inclusion of RIFIN reagents in a multivalent vaccine or therapies should be given a higher priority over a stand-alone target.

RIFINs interact directly with LILRB1 through specific amino acids or conformations within their C-terminal variable region. The binding is important for iRBC interaction with various inhibitory receptors expressed on leucocytes which contribute to the pathogenesis of severe malaria (2). In this study we did not observe differences in seroprevalence of antigens located to the N-terminal conserved vs. the polymorphic regions (Figure 2C), suggesting that both regions induce immunity at equivalent levels. RIFINs that are associated with reduced risk to febrile malaria were all drawn from the conserved N-terminal region (Table S2). This could suggest that clinical immunity is mediated by antibody responses against the conserved sections while the variable region, which induces a more robust response in infants, mediates protection against severe malaria, as suggested elsewhere (2). However, to draw a definitive conclusion on antibodies generated against the conserved or polymorphic regions, further analysis of each region will be required with samples drawn from a malaria exposed population, over different age groups and malaria outcomes.

The role of B-RIFINs as targets of protective immunity against clinical malaria remains unclear. Indeed, their role in merozoite invasion, sequestration, cytoadherence of infected erythrocytes, and involvement in the development of severe malaria requires further studies. However, with specific B-RIFINs variants such as PF3D7_1300600 showing localization on the surface of merozoites and gametocytes (20), and only a few RIFINs variants/hybrids having been studied, additional evaluation will be needed to fully grasp their functions. Alternatively, in the absence of direct exposure to host immunity during intraerythrocytic growth, the antigens may represent potential correlates of protective immunity and not necessarily as targets of the protective immunity.

We also assessed human acquisition of antibodies to SURFINs and STEVORs. In a recent study, a polyclonal antibody (anti- PF3D7_1254100) with broad specificity to different STEVOR variants localized STEVORs on the surface of non-permeabilized/non-fixed merozoites (53). Using this antibody, the authors showed that several STEVORs were also expressed in the intraerythrocytic stage of the parasite. Expression of STEVORs on the surface of merozoites and iRBC potentially changes the antigenic property of parasites thus creating antigenic diversity and contributing to immune escape. Expression of a glycophorin C binding STEVOR on the iRBC leads to PfEMP1-independent rosette formation which can be blocked by anti-STEVOR antibodies (26). Similarly, the antibodies effectively inhibit erythrocyte invasion of merozoites (26). Taken together, these studies illustrate the potential mode of action of antibodies against STEVORs in protective immunity against malaria.

The function of PF3D7_0113600 (SURFIN1.2) which was associated with reduced risk of febrile malaria selected in this study is unknown, but has been suggested to be involved in cell surface adhesion (54) and is associated with chloroquine sensitivity (55). However, the *surf1.2* gene is annotated as a pseudogene in the malaria genome database (www.PlasmoDB.org); hence *in vivo* expression in parasites must be confirmed before further analysis. Other SURFINs such as SURFIN4.2 have been putatively associated with erythrocyte invasion (54). Further understanding of the potential role of STEVOR and SURFIN proteins selected in the present analysis will be required.

For all RIFINs, SURFINs, and STEVORs, we did not observe any reduced chance of a protein being recognized by sera based on length, and comparisons between truncates of the same protein showed comparable immunoreactivities. The variable C-terminus were relatively more seroprevalent than the conserved N-terminal truncates (Figure 2C). In a similar manner, less conserved regions of PfMSP10 were recently observed to be more seroprevalent compared to the highly conserved N-terminal segment (56), and historically most naturally acquired antibodies to PfMSP1 recognize its variable domains (57). It's suggested that this differential reactivity could contribute to the parasite's host immune evasion mechanism, referred to as the smoke-screen strategy (58).

Although we detected potential correlates of protection in the population, we may have missed weak or rare immune associations or overestimated others due to the limited sample size assayed while analyzing multiple antigens over a wide age range. Another limitation was the absence of infection or clinical malaria historical data prior to conducting the prospective study, which could have reduced heterogeneity in samples and allowed more accurate statistical adjustments. The protein library used may also not accurately reflect the proteins expressed by the local parasite population causing the infection, due to its construction based upon the reference Pf3D7 parasite strain. In addition, some proteins may not have been optimally expressed as native parasite proteins, or may have lacked important epitopes, such as within or associated with surface exposed central hydrophobic regions or variable regions that were excluded during protein truncation to ease recombinant protein expression. Nevertheless, the high immunoreactivities observed in this study suggest that the expressed proteins were similar or shared conformations to native parasite proteins. Further studies are required to clarify the role of the selected antigens in protection from malaria pathogenesis.

In conclusion, the extensive global immunoreactivity of RIFINs, SURFINs, and STEVORs presented in this study may reflect a parasite strategy of expressing multiple VSAs in the blood stage, hence enhancing the likelihood of survival and establishment of chronic infections in the host. The identified four B-RIFIN antigens (PF3D7_0201000, PF3D7_1254500, PF3D7_1040600, PF3D7_1041100), STEVOR (PF3D7_0732000), and SURFIN 1.2 (PF3D7_0113600) may indicate important targets or correlates of protective immunity against febrile malaria that need further evaluation. The development of this protein library will facilitate high-throughput evaluation of protective immune profiles toward the identification and prioritization of potential *P. falciparum* vaccine antigens or correlates of clinical protection by interrogating human samples obtained from well-founded studies.

## Data Availability Statement

The datasets generated for this study are available on request to the corresponding author.

## Ethics Statement

The studies involving human participants were reviewed and approved by Institutional Research and Ethics Committee of Lacor Hospital (LHIREC 023/09/13), Uganda National Council for Science and Technology (HS1403) in Uganda, Ethics Committees of the Research Institute for Microbial Diseases, Osaka University and Ehime University. Written informed consent to participate in this study was provided by the participants and/or legal guardians/next of kin.

## Author Contributions

BK, TT, and ET conceived and designed experiments. BK, HN, NP, MW, MM, EN, BB, AY, TE, and TH conducted experiments. BK, MW, TT, and ET analyzed the data. BK, TT, and ET wrote the manuscript. All authors discussed and edited the manuscript.

## Conflict of Interest

The authors declare that the research was conducted in the absence of any commercial or financial relationships that could be construed as a potential conflict of interest.

## Abbreviations

RIFIN: Repetitive interspersed family
STEVOR: Subtelomeric variable open reading frame
SURFIN: Surface-associated interspersed gene family
WGCFS: Wheat germ cell-free system
PPE: Potential protective efficacy
VSA: Variable surface antigen
iRBCs: Infected red blood cells.
