# Supplementary material for: Global Repertoire of Human Antibodies Against Plasmodium falciparum RIFINs, SURFINs, and STEVORs in a Malaria Exposed Population
Source: Front Immunol. 2020 May 12;11:893. doi: 10.3389/fimmu.2020.00893 (PMC7235171; doi:10.3389/fimmu.2020.00893)
Supplement: Supplementary file 2 [file Data_Sheet_1.PDF]

Supplementary Figures

Global repertoire of human antibodies against *Plasmodium falciparum* RIFINs, SURFINs, and STEVORs in a malaria exposed population

Bernard N. Kanoi[1*], Hikaru Nagaoka[1], Michael T. White[2], Masayuki Morita[1], Nirianne M. Q. Palacpac[3], Edward H. Ntege[4], Betty Balikagala[5], Adoke Yeka[6], Thomas G. Egwang[7], Toshihiro Horii[3], Takafumi Tsuboi[1], Eizo Takashima[1*]

[1]*Division of Malaria Research, Proteo-Science Center, Ehime University, Matsuyama, Ehime 790-8577, Japan*
[2]*Department of Parasites and Insect Vectors, Pasteur Institute, Paris, France*
[3]*Department of Malaria Vaccine Development, Research Institute for Microbial Diseases, Osaka University, Suita, 565-0871, Japan*
[4]*Department of Plastic and Reconstructive Surgery, University of the Ryukyus, Graduate School of Medicine and Hospital, Okinawa, 903-0215, Japan*
[5]*Department of Tropical Medicine and Parasitology, School of Medicine, Juntendo University, Tokyo, 113-8421, Japan*
[6]*Makerere University School of Public Health, Kampala, Uganda*
[7]*Med Biotech Laboratories, Plot 4-6 Bell Close, Port Bell Road Luzira, Kampala, Uganda*

* Address correspondence to:
Eizo Takashima; takashima.eizo.mz@ehime-u.ac.jp
Bernard N. Kanoi: kanoi.bernard_nganga.vx@ehime-u.ac.jp
Tel.: +81-89-927-8277; fax: +81-89-927-8528.

Fig S1

A

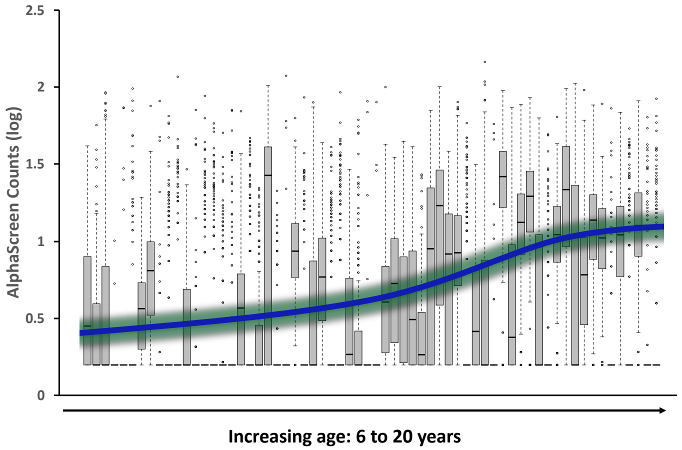

A. Overall seroreactivity of each individual showed an age dependent increasing trend. Box plots illustrate medians with 25th and 75th percentile and whiskers for 10th and 90th percentile of antibody responses to RIFINs, STEVORs, and SURFINs. The blue line indicates a trendline with confidence intervals presented as shadow.

B

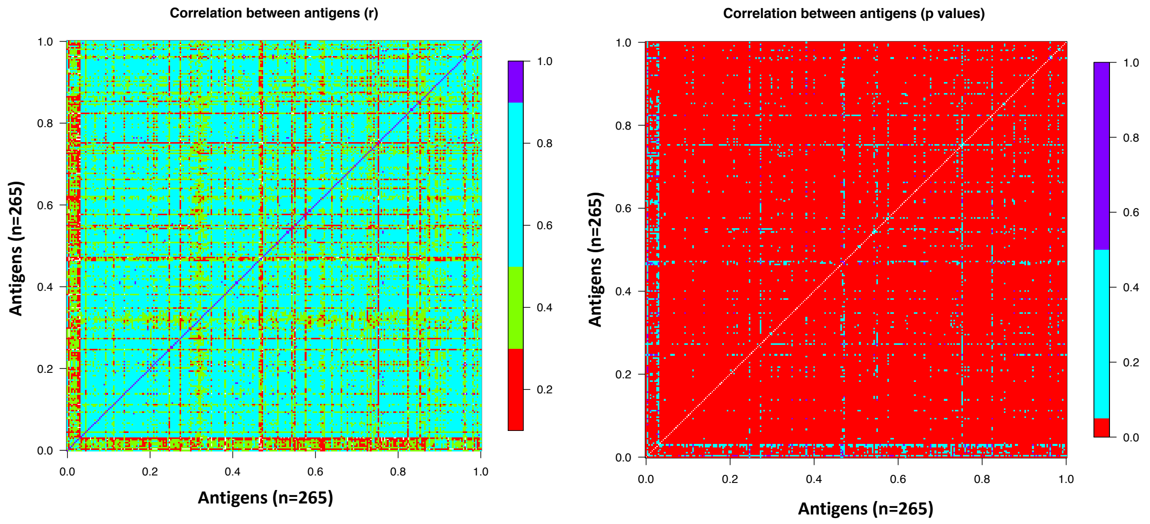

B. Antibody acquisition correlation matrix. The value score for the r correlation coefficients and the corresponding *P* values are shown on the right of each matrix.
